# The Incidence of Gastrointestinal Stromal Tumors in Obese Patients—A Large Single Center Experience

**DOI:** 10.3390/medicina57111242

**Published:** 2021-11-14

**Authors:** Natalia Dowgiałło-Gornowicz, Klaudia Sztaba, Paweł Lech, Anna Botulińska, Maciej Michalik

**Affiliations:** 1 Department of General, Minimally Invasive and Elderly Surgery, University of Warmia and Mazury, 10-045 Olsztyn, Poland; klaudia.sztaba@op.pl (K.S.); lechpawel@op.pl (P.L.); 2 Department of Family Medicine and Infectious Disease, University of Warmia and Mazury, 10-045 Olsztyn, Poland; annabotulinska@gmail.com; 3 Department of General, Colorectal and Oncological Surgery, Nicolaus Copernicus University, 85-168 Bydgoszcz, Poland; michs1@wp.pl

**Keywords:** GIST, gastrointestinal stromal tumor, laparoscopic sleeve gastrectomy, bariatric surgery

## Abstract

*Background and Objectives*: Gastrointestinal stromal tumors (GISTs) are rare mesenchymal neoplasms located mainly in the fundus (60–70%). The incidence of GIST is approximately 10 per million population per year in Europe, with a peak incidence at the age of 63. Recent studies suggest that morbidly obese patients have a higher incidence of GIST than the general population. The aim of this study was to analyze the incidence of GIST in patients undergoing laparoscopic sleeve gastrectomy (LSG) in our department. *Materials and Methods*: this paper present the retrospective study of prospectively collected data of 1564 patients who underwent LSG in a single large bariatric center from October 2013 to September 2021. After surgery, each sample of the resected stomach was sent for histopathological examination. For the analysis, we included patients diagnosed with GIST intraoperatively or postoperatively. *Results*: GISTs were found in five patients (0.31%). There were three men and two women. The mean age was 50.2 (range 32–63 ± 11.8) and the mean preoperative body mass index was 43.3 kg/m^2^ (40–49.4 ± 3.2). In four cases, GISTs were found in the fundus (80%), and in one in the pylorus (20%). None of the tumors were larger than 7 mm in diameter and all were diagnosed as a very low-risk category. No adjuvant treatment was required. All patients achieved good or satisfactory bariatric and metabolic results. *Conclusions*: The incidence of GIST in our study was estimated at 0.31%. All patients had a very low-risk GIST and no recurrence until follow-up. Recent literature suggests that the risk of GIST is higher in the obese population, and therefore surgeons should be aware of the risk of incidental GIST during LSG.

## 1. Introduction

Gastrointestinal stromal tumors (GISTs) are rare mesenchymal neoplasms located along the entire length of the gastrointestinal wall. GISTs probably originate from the interstitial cells of Cajal [1]. While Cajal cells are found mainly in the fundus, the stomach is also the most common location for GIST (60–70%) [2]. The prevalence of GIST ranges from 10 per million of the population annually in Europe to about 20 in Asia, with a peak incidence at age 63 [3]. GISTs represent a wide spectrum of symptoms, ranging from completely asymptomatic to gastrointestinal disorders and varying degrees of aggressiveness based on tumor location, size, and mitotic index [2,3].

According to Eurostat, in 2019, almost 20% of adult Europeans were obese [4]. About 45% of women and 60% of men were overweight [4]. In Poland, the statistics were worse. Furthermore, in recent decades, there has been a growing tendency towards being overweight. This is likely the reason for the development of bariatric and metabolic surgery worldwide. Currently, the most common bariatric procedure is laparoscopic sleeve gastrectomy (LSG) [5].

Since recent studies suggest that the prevalence of GISTs in morbidly obese people undergoing bariatric surgery is higher than in the general population, LSG seems to be a good and safe treatment for both conditions [6].

The aim of the study was to analyze the incidence of GISTs in patients undergoing LSG in our department. Second, we aimed to investigate whether obesity increases the risk of GIST by comparing our results with an overview of the literature.

## 2. Materials and Methods

This is the retrospective study of prospectively collected data of 1564 patients undergoing LSG in a single large bariatric center from October 2013 to September 2021. All patients were qualified for surgery in accordance with international recommendations. Preoperative examinations, such as ultrasound and gastroscopy, were performed for all patients. All operations were performed by the same team of bariatric surgeons, according to standardized technique [7]. After surgery, each stomach resection sample was transferred to the pathology department for histopathological examination. The analysis included patients diagnosed with GIST intraoperatively or postoperatively. We analyzed gender, age, BMI, and comorbidities. Tumor parameters such as size, location, type, and mitotic index were also studied. All patients underwent postoperative survey during personal or telephonic consultations. They were conducted one month after the surgery and every six months thereafter. Descriptive statistics were used. The median and standard deviation distributions were estimated. The differences in mean age and body mass index between patients with GISTs and controls were assessed using Student’s t-test. A *p*-value <0.05 was considered significant. The analysis was conducted using Statistica (data analysis software system), version 13. http://statistica.io (accessed on 1 October 2021) TIBCO Software Inc., Krakow, Poland (2017).

## 3. Results

A total of 1564 LSGs were performed in one surgical department from October 2013 to September 2021. All patients underwent preoperative endoscopic examination, and no pathology was found during the examination. After surgery all gastric samples were subjected to histopathological evaluation. GISTs were found in five patients (0.31%). There were three men and two women in the group. The mean age was 50.2 (range 32–63 ± 11.8), and the mean preoperative body mass index was 43.3 kg/m^2^ (40–49.4 ± 3.2), Table 1. Patients with GISTs were statistically significantly older than those in the control group, *p* = 0.03. There was no statistical difference in the preoperative body mass index, *p* = 0.15, Table 1.

There were no postoperative complications in this group of patients. The mean follow-up was 20.6 months (range 3–44 ± 17.2), and the mean percentage of excess weight loss (%EWL) was 55.7% (range 42.1–86 ± 15.8), as shown in Table 1. All patients obtained good or satisfactory results after the surgery according to BAROS [8]. Patient 2 stopped taking drugs for arterial hypertension in the first week after surgery and took reduced doses of drugs for diabetes mellitus in the first month after the surgery. Patient 5 discontinued drugs for arterial hypertension and diabetes mellitus in the first month after the surgery.

**Figure 1:**
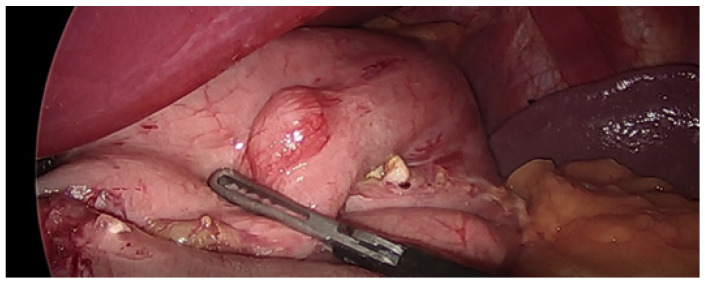
GIST in the fundus found intraoperatively in Patient 5. (White arrow indicates the tumor).

In four cases, GISTs were found in the fundus (80%), and in one was found in the pylorus (20%), Figure 1. No tumors were larger than 7 mm in diameter, as shown in Table 2. Pathologists found that all GISTs were diagnosed as a very low risk category. The results of the lesions are presented in Table 2. No adjuvant treatment was required, but patients remained under the control of the oncology department.

## 4. Discussion

In this retrospective single-center study, we estimated the incidence of GISTs during LSG to be 0.31%. In the latest meta-analysis of Wang et al., the overall incidence of GISTs in nearly 33.000 patients undergoing bariatric procedures was 0.45% (95% CI 0.31–0.61) [9]. Although the overall prevalence of GISTs is about 0.006–0.0015%, the incidence among obese patients is higher [10].

Obesity is a proven risk factor for many neoplasms, such as colorectal, breast, or endometrial cancers [11]. The major pathways in which obesity promotes tumorigenesis include hyperinsulinemia/insulin resistance and insulin-like growth factor-I (IGF-I) abnormalities; sex hormone biosynthesis and pathway; low-grade inflammation and oxidative stress; alterations in adipocytokines; factors deriving from ectopic fat deposition; microenvironment and cellular disorders; disruption of circadian rhythms and dietary nutrients; altered intestinal microbiome; and mechanic factors in obesity [11,12,13]. In addition, the authors noted a correlation between ghrelin and GISTs, both of which are mainly located in the fundus of stomach [14,15]. It may also suggest that GISTs are more frequently observed in obese patients than non-obese patients.

On the other hand, most small GISTs remain asymptomatic and undiagnosed [2]. Even when patients undergo gastroscopy, GISTs are often found on the serous side of the stomach without mucosal involvement, rendering them underdiagnosed [16]. However, if a change in the stomach is suspected, the diagnosis can be extended with endoscopic ultrasound [17]. Research shows that ultrasound fine-needle biopsy is a safe and effective tool, enabling early histopathological diagnostics and the treatment of small sub-epithelial lesions [17,18]. Interesting results have been reported by Chiappetta et al [19,20]. In the first release, the incidence of GISTs was 0.31% (2603 patients, 2010–2014) [19]. In the next study, it was 1.0% (1544 patients, 2014–2018) [20]. They assumed that paying more attention to such accidental discoveries would increase the incidence of GISTs during bariatric surgery. This is a remarkable observation, but our case series and previous reports have undeniably drawn attention to the higher incidence of GISTs in obese patients [21,22,23,24,25].

In our study, no incidentally resected tumors were larger than 7 mm with a low mitotic index, and all were defined as a very low-risk tumor according to Joensuu classification [26]. This is in line with previous reports from the review by Fernandez et al. [6]. A total of 96% of tumors were smaller than 2 cm, and 99% of GISTs have a low mitotic index [6]. According to the literature, 10% to 30% of GISTs can develop into a malignant clinical course, regardless of tumor size [17,27]. Moreover, Zemła et al. found in their retrospective analysis that body mass index did not correlate with a higher mitotic index [28]. All our GISTs were located extramurally, and their diagnoses were virtually impossible without surgery. The European Society for Medical Oncology recommends surgical resection for even a very low-risk GIST [29]. Considering the fact above, complete resection of the reported tumor is necessary to establish the diagnosis and in most cases, it is curable, which confirms the long-term results [22,23].

The strength of the study was the histopathological examination of each stomach sample, which eliminated the awareness of surgeons. Moreover, as far as we know, it is the second report conducted on this topic in this part of Europe [24].

This study has some limitations. It is a single-center retrospective study, and the diagnosis of GISTs depended on the pathology department. The follow-up was 3 to 44 months after the surgery, and patients are still under surveillance to rule out the disease recurrence.

## 5. Conclusions

The incidence of GISTs in our study was estimated at 0.31%. All patients had a very low-risk GIST and no recurrence until follow up. Recent literature suggests that the risk of GISTs is higher in the obese population; therefore, surgeons should be aware of the risk of incidental GISTs during a LSG.

## Figures and Tables

**Table 1:** Characteristics of patients with GISTs and controls. (BMI—body mass index; preop—preoperative; postop—postoperative; %EWL—percent of excess weight loss; SD—standard deviation).

	Patients with GISTs	Controls	*p*
Male	3 (60%)	357 (22.9%)	
Female	2 (40%)	1202 (77.1%)	
Age [years] (range ± SD)	50.2 (32–63 ± 11.8)	39.8 (18–71 ± 10.9)	0.03
BMI preop [kg/m^2^] (range ± SD)	43.3 (40–49.4 ± 3.2)	44.9 (38.2–76.8 ± 5.7)	0.15
BMI postop [kg/m^2^] (range ± SD)	31.6 (24.2–34.5 ± 3.7)	-	
%EWL [%] (range ± SD)	55.7 (42.1–86 ± 15.8)	-	
Follow up [months] (range ± SD)	20.6 (3–44 ± 17.2)	-	

**Table 2:** Characteristics of patients with histopathological results. (DM2—diabetes mellitus 2; AH—arterial hypertension; DCM—dilated cardiomyopathy; AF—atrial fibrillation; HT—hypothyroidism; BMI—body mass index; preop—preoperative; postop—postoperative; %EWL—percentage of excess weight loss).

Patient	1	2	3	4	5
**Sex**	Male	Male	Female	Female	Male
**Age [years]**	42	63	62	32	52
**Comorbidities**	none	DM2, AH, DCM, AF	none	HT	DM2, AH
**BMI preop [kg/m^2^]**	42.5	42.4	42.4	40	49.4
**BMI postop [kg/m^2^]**	32.6	34.2	32.7	24.2	34.5
**Date of surgery [mm-yyyy]**	June 2021	July 2021	February 2019	August 2019	August 2020
**GISTs characteristics**					
**Size [mm]**	6	3	7	4	7
**Localization**	Fundus	Fundus	Pylorus	Fundus	Fundus
**Type**	Spindle cell	Spindle cell	Spindle cell	Spindle cell	Spindle cell
**Mitotic rate**	1/5 mm^2^	1/5 mm^2^	1/5 mm^2^	1/5 mm^2^	1/5 mm^2^
**CD117**	+	+	+	+	+
**CD34**	+	+	+	+	+
**Vimentin**	-	-	+	-	-
**S-100**	-	-	-	-	-
**Ki-67**	+<1% cells	+<1% cells	+<2% cells	+<1% cells	+<1% cells
**Desmin**	-	-	-	-	-
**SMA**	-	-	-	-	-

## Data Availability

The data presented in this study are available on request from the corresponding author.
